# A Therapeutic Chemical Chaperone Inhibits Cholera Intoxication and Unfolding/Translocation of the Cholera Toxin A1 Subunit

**DOI:** 10.1371/journal.pone.0018825

**Published:** 2011-04-19

**Authors:** Michael Taylor, Tuhina Banerjee, Fernando Navarro-Garcia, Jazmin Huerta, Shane Massey, Mansfield Burlingame, Abhay H. Pande, Suren A. Tatulian, Ken Teter

**Affiliations:** 1 Burnett School of Biomedical Sciences, College of Medicine, University of Central Florida, Orlando, Florida, United States of America; 2 Department of Cell Biology, Centro de Investigación y de Estudios Avanzados del IPN (CINVESTAV-Zacatenco), México City, Mexico; 3 Department of Physics, University of Central Florida, Orlando, Florida, United States of America; Louisiana State University and A & M College, United States of America

## Abstract

Cholera toxin (CT) travels as an intact AB_5_ protein toxin from the cell surface to the endoplasmic reticulum (ER) of an intoxicated cell. In the ER, the catalytic A1 subunit dissociates from the rest of the toxin. Translocation of CTA1 from the ER to the cytosol is then facilitated by the quality control mechanism of ER-associated degradation (ERAD). Thermal instability in the isolated CTA1 subunit generates an unfolded toxin conformation that acts as the trigger for ERAD-mediated translocation to the cytosol. In this work, we show by circular dichroism and fluorescence spectroscopy that exposure to 4-phenylbutyric acid (PBA) inhibited the thermal unfolding of CTA1. This, in turn, blocked the ER-to-cytosol export of CTA1 and productive intoxication of either cultured cells or rat ileal loops. In cell culture studies PBA did not affect CT trafficking to the ER, CTA1 dissociation from the holotoxin, or functioning of the ERAD system. PBA is currently used as a therapeutic agent to treat urea cycle disorders. Our data suggest PBA could also be used in a new application to prevent or possibly treat cholera.

## Introduction

AB toxins consist of an enzymatic A subunit and a cell-binding B subunit [1]. These toxins are secreted into the extracellular milieu, but they act upon targets within the eukaryotic cytosol. The toxins must therefore cross a membrane barrier in order to function. Some AB toxins travel by vesicle carriers from the cell surface to the endoplasmic reticulum (ER) before passing into the cytosol [2]. These ER-translocating toxins enter the ER as intact holotoxins, but environmental conditions in the ER promote the dissociation of the catalytic subunit from the rest of the toxin. Translocation of the isolated A chain from the ER to the cytosol is then facilitated by the quality control mechanism of ER-associated degradation (ERAD) [3]. Exported ERAD substrates are normally targeted for ubiqutin-dependent proteasomal degradation, but the A chains of ER-translocating toxins have few lysine residues for ubiquitin conjugation and thus effectively avoid degradation by the 26S proteasome [4]–[7].

Cholera toxin (CT) is an AB_5_-type, ER-translocating toxin [8], [9]. Its A subunit is proteolytically nicked to generate a disulfide-linked A1/A2 heterodimer. The enzymatic A1 subunit dissociates from the rest of the toxin in the ER and enters the cytosol where it ADP-ribosylates the stimulatory α subunit of the heterotrimeric G protein (Gsα). Adenylate cyclase is activated by the ADP-ribosylated form of Gsα, which in turn leads to elevated levels of intracellular cAMP. A chloride channel, the cystic fibrosis transmembrane regulator, opens in response to the signaling events triggered by high cAMP levels. The osmotic movement of water which follows chloride efflux into the intestinal lumen generates the profuse watery diarrhea of cholera.

Thermal instability in the isolated CTA1 subunit serves as the trigger for ERAD-mediated translocation to the cytosol [10], [11]. CTA1 is held in a stable conformation by its association with CTA2/CTB_5_, but it unfolds spontaneously at physiological temperature when it is released from the rest of the toxin in the ER [11]–[13]. The loss of CTA1 tertiary structure that accompanies its dissociation from the holotoxin identifies CTA1 as a misfolded protein for ERAD processing [10]. After ERAD-mediated translocation to the cytosol, CTA1 interacts with ADP-ribosylation factors and possibly other host factors in order to regain a folded, active conformation [11], [14], [15].

Because of its central role in ERAD-mediated toxin translocation, CTA1 thermal instability represents a promising target for anti-toxin therapeutics. Inhibition of CTA1 unfolding in the ER would prevent its recognition by the ERAD system, its translocation to the cytosol, and, thus, its cytopathic effect. We recently used glycerol, a chemical chaperone that stabilizes protein structures and disrupts ERAD-substrate interactions, to provide proof-of-principle for this therapeutic strategy: glycerol treatment specifically stabilized the tertiary structure of CTA1, which in turn prevented CTA1 translocation to the cytosol and productive intoxication [10]. Acidic pH likewise prevented the thermal disordering of CTA1 tertiary structure and CTA1 translocation to the cytosol [16]. These results strongly suggest that cholera could be prevented or treated with therapeutic agents that stabilize the tertiary structure of CTA1.

The overall aim of this work was to determine if a therapeutic chemical chaperone could be used to block the cytopathic effects of CT. Here, we report that 4-phenylbutyric acid (PBA) inhibits the thermal unfolding of CTA1, the ER-to-cytosol translocation of CTA1, and CT intoxication. PBA is a chemical chaperone and a therapeutic agent approved by the Food and Drug Administration (FDA) for the management of urea cycle disorders [17], [18]. The therapeutic value of PBA in treating these disorders relates to its function as an ammonia scavenger rather than its ability to function as a chemical chaperone. In vitro, PBA bound to the CT holotoxin and the CTA1 polypeptide with nM affinity but did not bind to the CTB pentamer. In vivo, PBA effectively blocked fluid accumulation in the physiological ileal loop model of CT intoxication. PBA could thus represent a novel therapeutic agent for the prevention or treatment of cholera.

## Results

### PBA binds directly to CT and CTA1

**Figure 1:**
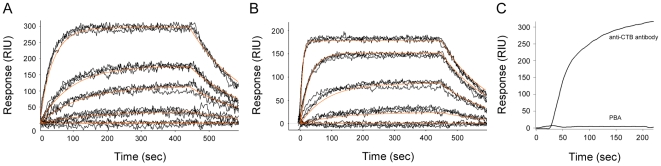
PBA binds to CT and CTA1 but not to CTB_5_. PBA was perfused over SPR sensor slides that had been coated with CT (A), CTA1 (B), or CTB_5_ (C). PBA concentrations of 0.1, 1, 10, 100, and 1000 µM were used in panels A and B; 100 µM PBA was used for panel C. As a positive control, an anti-CTB antibody was also perfused over the sensor slide in panel C. PBA was removed from the perfusion buffer either 390 sec (A and B) or 130 sec (C) into the experiment. In panels A and B, measurements collected from three independent experiments are shown. The orange lines represent best fit curves for the presented data.

To determine if PBA binds directly to CTA1, we used the method of surface plasmon resonance (SPR). CT, CTA1, and the CTB pentamer were each appended to separate SPR sensor slides. Ligand binding to a toxin-coated sensor slide increases the mass on the slide, and this in turn generates a change in the resonance angle of the reflected light which is recorded as the refractive index [19]. An increase in the refractive index unit (RIU) was detected when PBA was perfused over the CT and CTA1 sensor slides (Figs. 1A–B). However, no change in the RIU was recorded when 100 µM PBA was perfused over the CTB_5_ sensor slide (Fig. 1C). A strong positive signal was obtained when an anti-CTB antibody was perfused over the CTB_5_ sensor slide, thus confirming the presence of CTB_5_ on our plate. These results demonstrated that PBA binds directly to CTA1 and strongly suggested that PBA binding to the CT holotoxin is mediated by the A1 subunit rather than the B pentamer.

The SPR experiments of Figures 1A–B were used to determine the binding affinity of PBA for CT and CTA1. As shown in Table 1, an equilibrium dissociation constant of about 10 nM was calculated for the interaction between PBA and either CT or CTA1. Further analysis of the SPR data indicated that 18 molecules of PBA bind to each molecule of CTA1. The high affinity interaction between PBA and CT/CTA1 suggested that PBA could serve as an anti-toxin therapeutic agent.

**Table 1:** Binding affinities between PBA and CT or CTA1.

Binding Parameter	k_a_ (1/Ms)	k_d_ (1/s)	K_D_ (nM)
PBA+CT	1.21×10^5^	1.38×10^−3^	11
PBA+CTA1	1.55×10^5^	1.31×10^−3^	9

### PBA inhibits the thermal unfolding of CTA1

**Figure 2:**
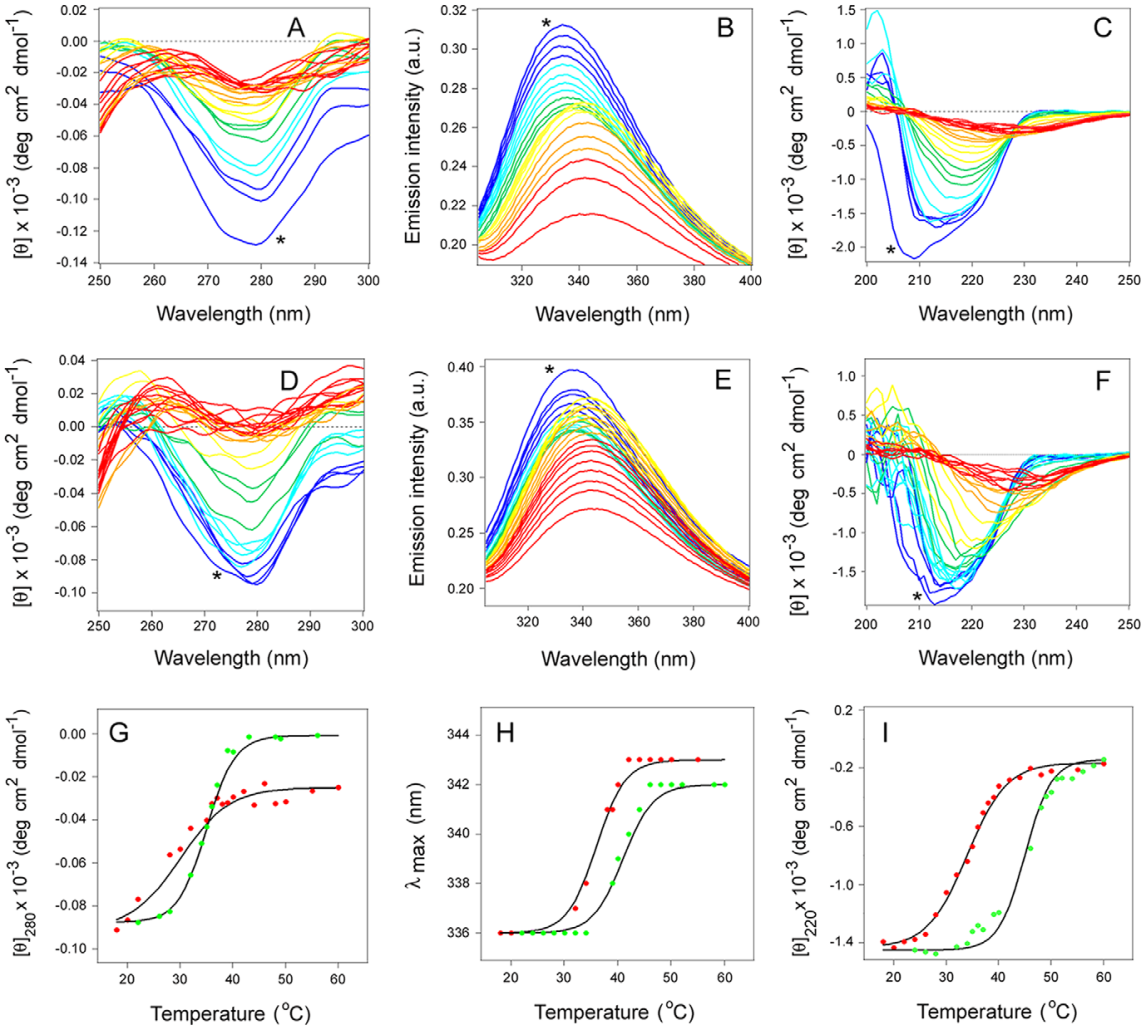
PBA inhibits the thermal unfolding of reduced CTA1/CTA2. The temperature-induced unfolding of a reduced CTA1/CTA2 heterodimer in the absence (A–C) or presence (D–F) of 100 µM PBA was monitored by near-UV CD (A, D), fluorescence spectroscopy (B, E), and far-UV CD (C, F). A final concentration of 10 mM β-ME was added to the buffer after an initial 18°C measurement was taken with the disulfide-linked CTA1/CTA2 heterodimer, as marked with asterisks. The change in color from blue to red corresponds to a change in temperature from 18°C to 60°C. Thermal unfolding profiles for the reduced CTA1/CTA2 heterodimer in the absence (red) or presence (green) of 100 µM PBA were derived from the data presented in panels A–F. (G): For near-UV CD analysis, the mean residue molar ellipticities at 280 nm ([θ]_280_) were plotted as a function of temperature. (H): For fluorescence spectroscopy, the maximum emission wavelength (λ_max_) was plotted as a function of temperature. (I): For far-UV CD analysis, the mean residue molar ellipticities at 220 nm ([θ]_220_) were plotted as a function of temperature.

Circular dichroism (CD) and fluorescence spectroscopy were used to examine the effect of PBA on CTA1 thermal stability. Measurements were taken on a reduced CTA1/CTA2 heterodimer during a step-wise increase in temperature from 18°C to 60°C. The 22 kDa CTA1 subunit is much larger than the 5 kDa CTA2 subunit, so it makes a major contribution to the CD spectra. Furthermore, CTA1 contains all three of the tryptophan residues that contributed to the fluorescence emission spectra. Reduction of the CTA1/CTA2 disulfide bond occurred when a final concentration of 10 mM β-mercaptoethanol (β-ME) was added to the toxin sample. This step was performed in order to mimic the holotoxin disassembly event that occurs in the ER [20]–[22]. Previous work has shown that complete reductive separation of CTA1 from CTA2 occurs within 1 minute of β-ME addition [11]. Control experiments further confirmed that PBA did not prevent the reductive separation of CTA1 from CTA2 (Fig. S1). The covalent association of CTA1 with CTA2 provides a degree of conformational stability to CTA1 [11], [12], so a shift in the CD spectra of the disulfide-linked CTA1/CTA2 heterodimer was apparent after β-ME addition at 18°C (Fig. 2A, C). This spectral shift did not occur for the PBA-treated toxin (Fig. 2D, F), which provided preliminary evidence for the stabilizing influence of PBA on the structure of CTA1.

By near-UV CD, we recorded a tertiary structure transition temperature (*T*_m_; the midpoint of transition between folded and unfolded conformations) of 30°C for reduced CTA1/CTA2 (Fig. 2A, G) and a *T*_m_ of 36°C for reduced and PBA-treated CTA1/CTA2 (Fig. 2D, G). Fluorescence spectroscopy documented a red shift to the maximum emission wavelength (λ_max_) of tryptophan fluorescence with a *T*_m_ of 36°C for CTA1 (Fig. 2B, H) and a *T*_m_ of 41°C for PBA-treated CTA1 (Fig. 2E, H). As assessed by far-UV CD, the toxin secondary structure exhibited a *T*_m_ of 35°C for reduced CTA1/CTA2 (Fig. 2C, I) and a *T*_m_ of 44°C for reduced and PBA-treated CTA1/CTA2 (Fig. 2F, I). These results demonstrated that PBA inhibits the thermal perturbation of both CTA1 secondary and tertiary structures.

CD and fluorescence spectroscopy measurements were also conducted with reduced CTA1/CTA2 heterodimers incubated in the presence of 1 or 10 µM PBA. The *T*_m_ values derived from all of our biophysical experiments are presented in Table 2, which documents the dose-dependent stabilization of CTA1 by PBA: although PBA conferred some thermal stability to the secondary and tertiary structures of CTA1 at 1 and 10 µM concentrations, the maximal stabilization of CTA1 was obtained with 100 µM PBA. Thus, at all tested concentrations, PBA provided some degree of conformational stability to the reduced CTA1 subunit.

**Table 2:** Dose-dependent thermal stabilization of CTA1 by PBA.

	Transition Temperature (°C)		
µM PBA	Near-UV CD	Fluorescence Spectroscopy	Far-UV CD
0	30.0	36.0	35.0
1	33.0	37.5	37.0
10	35.0	38.5	39.0
100	36.0	41.0	44.0

Previous work has suggested that the thermal unfolding of CTA1 begins with a localized loss of structure in the C-terminal A1_3_ subdomain [16]. To determine if this region was involved with the stabilizing effect of PBA on CTA1 structure, we repeated our biophysical experiments with a His-tagged CTA1 construct lacking the A1_3_ subdomain [23]. In contrast to the results obtained with a reduced CTA1/CTA2 heterodimer (Fig. 2) or with a full-length His-tagged CTA1 construct (Fig. S2), PBA did not alter the thermal unfolding profiles for the secondary and tertiary structures of the truncated CTA1 construct (Fig. S3). Thus, PBA appeared to stabilize CTA1 by preventing the initial temperature-induced loss of structure in the C-terminal A1_3_ subdomain.

**Figure 3:**
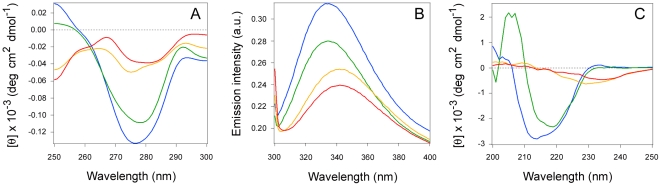
PBA does not promote the refolding of a thermally disordered CTA1 subunit. Near-UV CD (A), fluorescence spectroscopy (B), and far-UV CD (C) measurements of the CTA1/CTA2 heterodimer were taken at 18°C (blue spectra), 18°C after the addition of 10 mM β-ME (green spectra), after heating reduced CTA1/CTA2 to 37°C (yellow spectra), and one hour after the addition of 100 µM PBA to reduced CTA1/CTA2 at 37°C (red spectra). The substantial decrease in fluorescence intensity that occurred after an hour at 37°C in the presence of PBA likely resulted from the inherent temperature dependence of tryptophan fluorescence [50]. Notably, the λ_max_ red shift which accompanied the heating of reduced CTA1/CTA2 to 37°C was not reversed by the subsequent addition of PBA at 37°C.

Additional CD and fluorescence spectroscopy measurements were performed in order to determine whether PBA could induce unfolded CTA1 to regain its initial conformation. For these experiments, the reduced CTA1/CTA2 heterodimer was heated to 37°C. Near-UV CD, fluorescence spectroscopy, and far-UV CD were then used to assess the conformational state of reduced CTA1/CTA2 before and after the addition of 100 µM PBA. As shown in Figure 3, PBA did not promote the refolding of a thermally disordered CTA1 subunit. No significant changes to the structure of CTA1 occurred five minutes (not shown) or one hour after the addition of PBA at 37°C. Cooling CTA1 from 37°C to 18°C will allow the toxin to regain a folded conformation (Fig. S4), so the unfolding of CTA1 at 37°C is a reversible process. Thus, PBA will stabilize the folded CTA1 subunit but will not facilitate the renaturation of an unfolded CTA1 subunit.

### PBA inhibits CTA1 translocation to the cytosol

**Figure 4:**
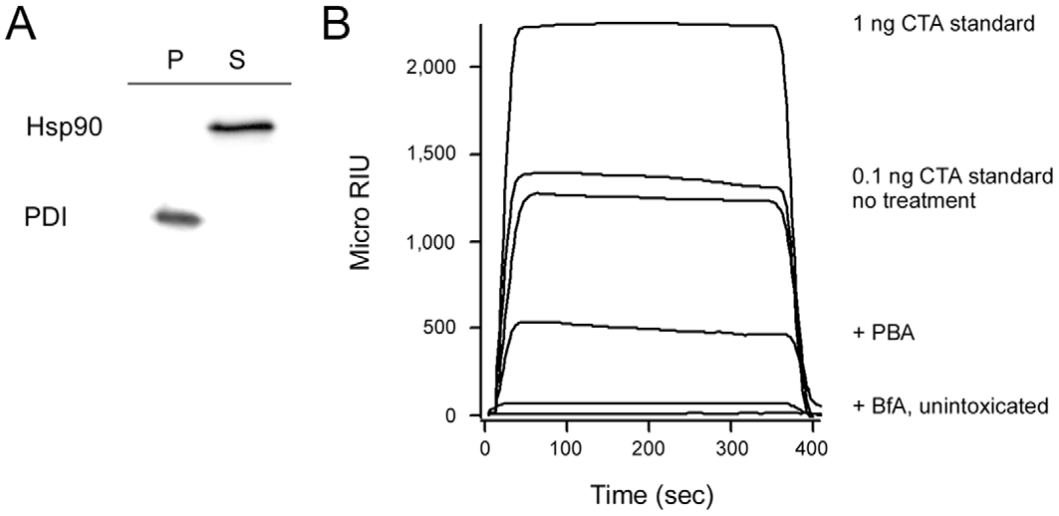
PBA blocks CTA1 translocation to the cytosol. HeLa cells were pulse-labeled at 4°C for 30 min with 1 µg/ml of CT. The cells were then chased for 2 hr at 37°C in toxin-free medium that contained no additions, 100 µM PBA, or 5 µg/ml of BfA. Selective permeabilization of the plasma membrane with digitonin was used to partition cell extracts into separate membrane (pellet; P) and cytosolic (supernatant; S) fractions. (A): Both fractions were probed by Western blot to establish the distributions of cytosolic marker Hsp90 and ER marker PDI. (B): An SPR sensor slide coated with an anti-CTA antibody was used to detect the cytosolic pool of CTA1 from untreated (no treatment) or drug-treated cells. CTA standards (100, 10, 1, and 0.1 ng/ml) were perfused over the sensor slide as positive controls; only the 1 and 0.1 ng/ml standards are shown for scaling purposes. A cytosolic fraction from unintoxicated cells was also generated for this experiment. One of three representative experiments is shown. At the end of each experiment, bound sample was stripped from the sensor slide.

Our collective data suggested that PBA can bind to holotoxin-associated CTA1 and can then prevent the spontaneous thermal unfolding of the dissociated CTA1 subunit. By our model, CTA1 stabilization would prevent its recognition by the ERAD system and its ERAD-mediated translocation to the cytosol. To test this prediction, we performed a translocation assay to monitor the ER-to-cytosol export of CTA1 in the absence or presence of PBA (Fig. 4). Surface-bound CT was chased into HeLa cells for two hours before organelle and cytosolic fractions were generated from the intoxicated cells. Western blot controls demonstrated that protein disulfide isomerase (PDI), a soluble ER resident protein, was found only in the pellet fraction which contained intact membrane-bound organelles (Fig. 4A). As expected, the cytosolic protein Hsp90 was found in the supernatant fraction which contained the cytosol (Fig. 4A). Our protocol could thus effectively segregate the cell extracts into distinct organelle and cytosolic fractions.

Only a minor pool of surface-bound CT (∼5%) is transported to the ER; the majority of internalized toxin is instead degraded in the lysosomes [24], [25]. We accordingly used the highly sensitive method of SPR to detect CTA1 in the cytosolic fractions from untreated and PBA-treated cells (Fig. 4B). For this experiment, SPR sensor slides were coated with an anti-CTA antibody. The cytosolic fractions from our cell extracts were then perfused over a sensor slide in order to detect the translocated, cytosolic pool of CTA1. No signal was obtained from the cytosol of unintoxicated cells or from the cytosol of cells intoxicated in the presence of brefeldin A (BfA), a drug that blocks toxin transport to the ER translocation site [24], [25]. In contrast, CTA1 was consistently detected in the cytosol of untreated HeLa cells after 2 hours of toxin internalization (*n* = 3). The cytosol from PBA-treated cells produced a weak SPR signal that was substantially attenuated in comparison to the signal obtained from the untreated control cells. These collective results indicated that BfA completely inhibited, and PBA partially inhibited, the delivery of CTA1 to the cytosol.

The association rate constant (k_a_) derived from SPR data is proportional to ligand concentration [19], so we calculated the levels of cytosolic CTA1 from a standard curve of the k_a_ values for the CTA standards (Fig. S5). With this method, we estimated that 3-fold less CTA1 was in the cytosol of PBA-treated cells than in the cytosol of untreated control cells. Thus, PBA effectively prevented CTA1 from reaching the cytosol of intoxicated cells.

### PBA does not inhibit retrograde toxin trafficking to the ER, PDI function, or ERAD activity

**Figure 5:**
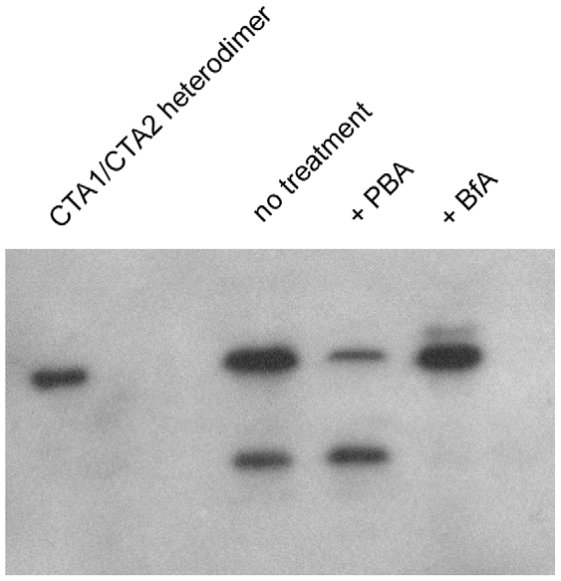
PBA does not block reduction of the CTA1/CTA2 disulfide bond in the ER. HeLa cells pulse-labeled at 4°C with recombinant CT were chased for 2 hr at 37°C in medium containing no additions (no treatment), 100 µM PBA, or 5 µg/ml of BfA. Membrane fractions from digitonin-permeabilized cells were resolved by non-reducing SDS-PAGE and probed by Western blot with an anti-CTA antibody. A purified, disulfide-linked CTA1/CTA2 heterodimer was also run as a control. We consistently detected slightly more reduced CTA1 in PBA-treated cells than in the untreated control cells (*n* = 4).

Control experiments were performed to ensure that PBA did not affect host-toxin interactions occurring before the translocation event. CT-treated HeLa cells were processed as described for the aforementioned SPR-based translocation assay, except in this case the pellet/organelle fractions were resolved by non-reducing SDS-PAGE and probed by Western blot with an anti-CTA antibody. As shown in Figure 5, reduced CTA1 was recovered from PBA-treated cells but not from BfA-treated cells. Reduction of the CTA1/CTA2 disulfide bond occurs in the ER [20], [21], so the presence of reduced CTA1 is indicative of CT transport to the ER. This experiment therefore demonstrated that, as expected, BfA prevented toxin trafficking to the ER. In contrast, PBA did not block CT transport from the cell surface to the ER. In fact, we consistently detected slightly more reduced CTA1 in PBA-treated cells than in the untreated control cells (*n* = 4). This indicated that CTA1 accumulated in the ER of PBA-treated cells, which was consistent with the PBA-induced block of CTA1 translocation (Fig. 4) and CTA1 secretion (Fig. S6).

**Figure 6:**
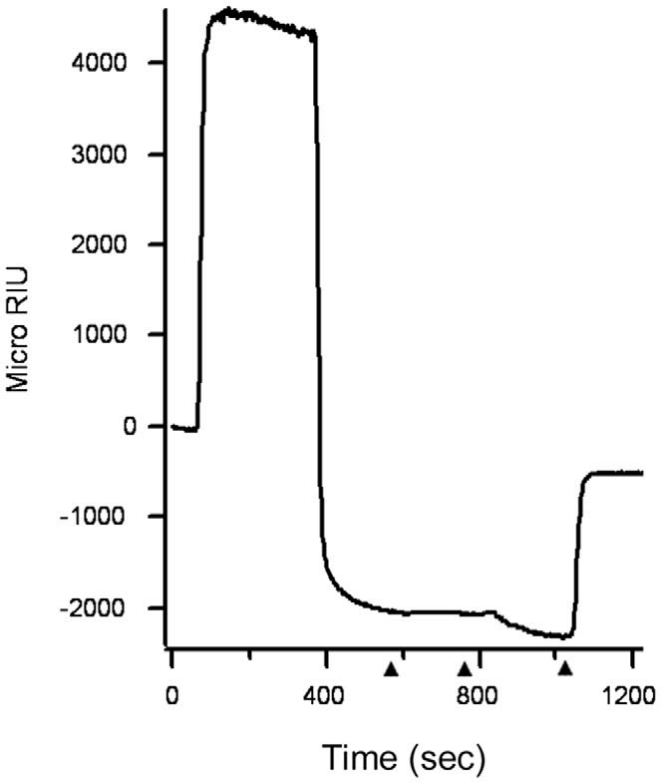
PBA does not block the dissociation of CTA1 from CTA2/CTB_5_. After appending CT to a GM1-coated SPR sensor slide, a baseline measurement corresponding to the mass of the holotoxin was recorded. Reduced PDI was then perfused over the slide in the presence of 100 µM PBA. PDI was present in the perfusion buffer until approximately 500 sec into the experiment, after which it was replaced with sequential additions of an anti-PDI antibody, an anti-CTA antibody, and an anti-CTB antibody (arrowheads). One of two representative experiments is shown.

Reduction of the CTA1/CTA2 disulfide bond is not sufficient for holotoxin disassembly; reduced CTA1 must be removed from CTA2/CTB_5_ by PDI [22]. To determine whether the PBA-induced block of CTA1 translocation was due to an inhibition of holotoxin disassembly, we used SPR to detect the PDI-mediated dissociation of CTA1 from CTA2/CTB_5_ (Fig. 6). When reduced PDI was perfused over a CT-coated SPR sensor slide, an increase in the RIU was followed by a precipitous drop in signal below the baseline value which represented the mass of the CT holotoxin. This indicated that the initial binding of PDI to the CT holotoxin quickly resulted in the loss of both PDI and a component of the holotoxin, most likely CTA1, from the sensor slide. Sequential additions of anti-PDI and anti-CTA antibodies to the sensor slide confirmed this interpretation, as neither antibody bound to the PDI-treated toxin. In contrast, the B pentamer remained on the slide as demonstrated by detection with an anti-CTB antibody. Since this experiment was performed with 100 µM PBA present in the perfusion buffer, we concluded that PBA binding to the CT holotoxin does not prevent the chaperone-assisted dissociation of CTA1 from CTA2/CTB_5_.

**Figure 7:**
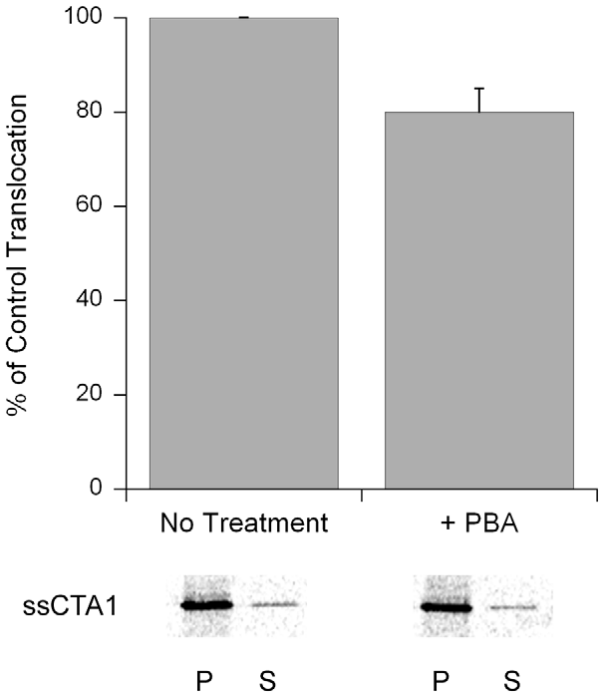
PBA does not inhibit the translocation of an ER-localized CTA1 construct. CHO cells were transfected with a plasmid encoding a CTA1 subunit appended with an ER-targeting signal sequence. Permeabilization of the plasma membrane with digitonin was used to partition cell extracts into separate organelle (pellet; P) and cytosolic (supernatant; S) fractions. The distribution of CTA1 immunoprecipitated from transfected cells after a 1 hr radiolabeling was visualized and quantified by SDS-PAGE with PhosphorImager analysis. Experiments were performed with untreated cells and cells exposed to 100 µM PBA. The means ± standard errors of the means of four independent experiments are presented in the graph.

To ensure that PBA did not induce a general block of ERAD activity, we performed another translocation assay with plasmid-expressed CTA1. This CTA1 construct contains an N-terminal signal sequence for co-translational insertion into the ER lumen [23]. Previous work has demonstrated that the entire detectable pool of expressed CTA1 is initially delivered to the ER where the signal sequence is removed [26]. The ER-localized toxin is then translocated back into the cytosol. We predicted that the ER-to-cytosol export of plasmid-expressed CTA1 would not be affected by PBA treatment because (i) co-translational insertion into the ER involves unfolded protein conformations; (ii) the incubation temperature of 37°C would prevent CTA1 from attaining a folded state after insertion into the ER; and (iii) PBA does not induce unfolded CTA1 to assume its native conformation (Fig. 3). Plasmid-expressed CTA1 would thus enter the ER in an unfolded state and would retain that conformation even in the presence of PBA, thereby promoting its ERAD-mediated translocation to the cytosol. As shown in Fig. 7, nearly equivalent amounts of plasmid-expressed CTA1 were exported to the cytosol of either untreated or PBA-treated cells. This result indicated that PBA does not block overall ERAD activity, but specifically inhibits the toxin-ERAD interaction that occurs with exogenously applied CT holotoxin. This specific inhibition most likely involves PBA-mediated stabilization of the folded CTA1 conformation which initially enters the ER as part of the CT holotoxin.

The control experiments presented in Figures 5, 6, 7 collectively demonstrated that PBA does not substantially inhibit toxin trafficking to the ER, toxin disassembly in the ER, or overall ERAD activity. PBA thus appeared to specifically block toxin entry into the cytosol at the level of ER-to-cytosol translocation.

### PBA blocks CT intoxication of cultured cells and ileal loops

**Figure 8:**
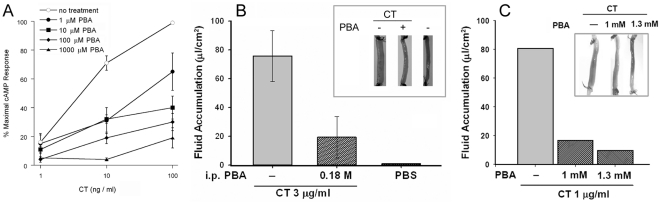
PBA inhibits CT intoxication. (A): HeLa cells were incubated with CT in the absence or presence of the stated PBA concentrations. After 2 hr of continual toxin exposure, toxicity was assessed from the elevated levels of intracellular cAMP. The means ± standard errors of the means of four independent experiments with triplicate samples are shown. (B): PBA was injected into the rat peritoneal cavity at a concentration of 0.18 M, which corresponded to 200 mg PBA/kg body weight. After 40 min, surgically sealed sections of intestine from mock-treated or PBA-treated rats were injected with 3 µg/ml of CT. An additional mock-treated rat was injected with PBS alone. Morphological examination (inset) and the calculation of fluid accumulation were performed 7 hr after toxin challenge. Results presented in the graph represent the averages ± standard deviations of data obtained from 4 rats per condition. (C): Ileal loops were injected with 1 µg/ml of CT or co-injected with CT and either 1 or 1.3 mM PBA. The evaluation of intoxication was performed as described in panel (B).

The PBA-induced inhibition of toxin translocation would prevent CTA1 from entering the cytosol where its Gsα target is located. PBA should therefore inhibit the cytopathic effects of CT. To determine the inhibitory effect of PBA on CT intoxication, we monitored cAMP levels in HeLa cells challenged with varying concentrations of CT in the absence or presence of PBA (Fig. 8A). A half-maximal effective concentration (EC_50_) of 4 ng CT/ml was calculated for cells exposed to toxin alone. In contrast, PBA-treated cells were highly resistant to CT. At the EC_50_, cells exposed to just 1 µM PBA were 10-fold more resistant to CT than the untreated control cells. Intoxicated cells treated with 10 µM PBA did not reach the half-maximal cAMP value of the control cells. Even at the highest toxin concentration of 100 ng/ml, cells treated with 10 µM PBA only produced 40% of the maximal cAMP signal obtained from the control cells. Cells treated with 10 µM PBA therefore required at least 25-fold higher concentrations of toxin to reach the EC_50_ obtained for the untreated control cells. Dose-dependent disruptions to CT intoxication were also recorded for cells exposed to 100 or 1000 µM PBA. Additional control experiments demonstrated that PBA did not inhibit the forskolin-induced elevation of intracellular cAMP: cells treated with 100 µM PBA and forskolin produced 97% of the cAMP levels recorded for cells treated with forskolin alone. Forskolin activates adenylate cyclase without the input of Gsα, so this observation demonstrated that PBA did not directly inhibit the production of cAMP by adenylate cyclase. Thus, PBA provided strong protection against CT in a cell culture system.

To examine the therapeutic potential of PBA as an anti-CT agent, we employed a physiological ileal loop model of CT intoxication. Rats were injected intraperitoneally with a concentration of PBA that has previously been used in animal studies: 200 mg PBA/kg body weight [27]–[29]. CT was injected directly into the ileal loop 40 minutes after the intraperitoneal injection of PBA, and the effects of intoxication were assessed seven hours after toxin challenge. As shown in Figure 8B, the intestinal distension and fluid accumulation induced by CT was dramatically attenuated in PBA-treated rats. The effects of intoxication were also substantially reduced when PBA and CT were co-injected directly into the intestinal loops (Fig. 8C). For both methods of drug delivery, CT-induced fluid accumulation was reduced by about 75% in comparison to control rats that were exposed to CT but not PBA. These results demonstrated that PBA, an FDA-approved therapeutic, can provide substantial in vivo protection against CT.

## Discussion

PBA is an FDA-approved drug for the treatment of urea cycle disorders [18]. The therapeutic value of PBA in treating these disorders relates to its function as an ammonia scavenger. However, PBA is also a chemical chaperone that can stabilize protein structures. In this capacity, PBA has been evaluated as a potential therapeutic for the treatment of many genetic diseases involving the ERAD-mediated degradation of mutant proteins that appear misfolded yet retain at least some wild-type function [17]. The PBA-mediated stabilization of these mutant proteins would, in theory, disrupt ERAD-substrate interactions and consequently result in the secretion of a functional protein. The release of a previously missing protein would correct the phenotype of the genetic defect and thus cure the disease. Since accumulating evidence suggests that CTA1 is processed as a typical ERAD substrate, we took a conceptually similar approach with CT: the PBA-mediated stabilization of CTA1 was predicted to disrupt ERAD-substrate interactions and thereby prevent the ERAD-mediated disease state. This strategy views cholera as a unique type of protein folding disease and suggests a new, anti-toxin application for a current clinical reagent.

At a concentration of 100 µM, PBA inhibited the thermal unfolding of CTA1, the ER-to-cytosol export of CTA1, and CT intoxication of cultured cells. Some inhibitory effects on CTA1 unfolding and CT intoxication were also observed with 1 and 10 µM PBA. Retrograde toxin trafficking to the ER, holotoxin disassembly by PDI, and ERAD activity in general were not altered by 100 µM PBA. However, we found that the stabilized pool of CTA1 was not efficiently secreted from PBA-treated cells. In contrast, we previously reported that glycerol-treated cells secreted more CTA1 than untreated control cells [10]. The different effects of PBA and glycerol on CTA1 secretion likely relates to their distinct modes of protein stabilization: PBA physically binds to the protein whereas glycerol forms a hydration shell around the protein [30]. The additional mass resulting from PBA interaction could possibly interfere with the packaging of stabilized CTA1 into carrier vesicles for secretory transport. Alternatively, different conformations for glycerol-stabilized CTA1 vs. PBA-stabilized CTA1 could promote toxin secretion from only glycerol-treated cells. The retention of PBA-stabilized CTA1 within the endomembrane system did not reduce cell viability over a 48 hour period as assessed by MTT assay (*n* = 2), possibly because the low levels of CT that traffic to the ER are not sufficient to overwhelm the transport and quality control functions of the secretory pathway.

PBA alters the expression of cytosolic chaperones, modulates the unfolded protein response, and inhibits chloride efflux from the cystic fibrosis transmembrane regulator. Each of these effects could influence host-CT interactions, but the concentration and duration of PBA treatment used in our studies is insufficient to elicit these off-target effects. We documented the block of CTA1 translocation and CT intoxication with two hour cell culture assays utilizing 100 µM PBA. In contrast, PBA-induced alterations to chaperone expression require a 48 hour exposure to at least 0.5 mM PBA [31], [32]. Alterations to the unfolded protein response require a 24 hour exposure to 10 mM PBA [33] or a 72 hour exposure to 2 mM PBA [34]. Inhibition of the cystic fibrosis transmembrane regulator in polarized epithelial cells only occurs after a 24 hour exposure to 5 mM PBA [35]. The low concentration of PBA required to stabilize CTA1, combined with the specific interaction between PBA and CTA1 (i.e., no PBA binding to CTB_5_ and no general effect on ERAD), thus minimizes off-target effects while still providing substantial cellular resistance to CT.

Exposure to 100 µM PBA generated a 3-fold reduction in the efficiency of CTA1 translocation to the cytosol. This, in turn, promoted a greater than 25-fold level of cellular resistance to CT. PBA-induced toxin resistance was also demonstrated in the physiological ileal loop model of intoxication. Previous work has shown that high levels of toxin resistance can be achieved without a complete block of toxin access to the cytosol [10], [36]–[38]. Furthermore, as recently shown for the plant toxin ricin, moderate levels of drug-induced toxin resistance in cell culture can correspond to high levels of drug-induced toxin resistance in an animal model [39]. We therefore expect that PBA will provide substantial protection against *V. cholerae* in an animal model of infection.

The maximum FDA-approved dosage of PBA is 20 g/day, which in clinical studies represented 10.6 to 13.8 g/M^2^/d. The drug was administered orally three to four times a day in the form of 500 mg tablets. With this regimen, patients attained serum PBA concentrations of 600–1,700 µM [40], [41]. It should therefore be possible to orally administer, at dosages well below the FDA-approved limit, a concentration of PBA that can inhibit CTA1 unfolding and CT intoxication in intestinal epithelial cells.

One limitation of a PBA-based therapeutic strategy is the timing of the inhibitory effect: PBA must act on the holotoxin before dissociation and translocation of the CTA1 subunit. While this would be acceptable for a preventative measure, its utility in treating patients already exposed to CT could be more limited. However, a number of observations suggest a post-exposure inhibition of CT might at least attenuate the effects of cholera. The rapid turnover of ADP-ribosylated Gsα [42] and the removal of ADP-ribose modifications by ADP-ribosyl(arginine)protein hydrolase [43] suggest that the activated form of Gsα can be cleared from the intoxicated cell, provided that CTA1 is also removed from the cytosol. Experiments demonstrating a correlation between toxin persistence in the cytosol and the extent of intoxication further support this possibility: the diminished potency of CT and recombinant CT constructs which are rapidly degraded in the cytosol indicates the cytosolic pool of toxin must be maintained at a certain level for optimal intoxication [6], [37], [44]. This precedent has been established for other virulence factors such as Shiga toxin [45] and the SpvB effector protein from Salmonella [46]. Thus, PBA application after the initial exposure to *Vibrio cholerae*/CT would prevent additional toxin from reaching the cytosol of an intoxicated cell, and this effect might attenuate the effects of intoxication. Attenuation of the disease state could also occur by the protective effect PBA would extend to newly differentiated enterocytes in the intestinal epithelium of an infected individual.

Our collective data indicates that PBA binds to the holotoxin-associated CTA1 subunit. The PBA-bound CTA1 subunit does not undergo thermal unfolding after toxin disassembly in the ER, and, as such, does not active the ERAD system. Toxin translocation to the cytosol is consequently blocked, which results in cellular protection against CT. PBA represents a promising therapeutic to prevent or possibly treat cholera because (i) it is already approved for human use; (ii) it is effective against CT at concentrations that can be attained in patients; (iii) it exhibits low nM affinity for CT/CTA1; and (iv) it inhibits CT in the physiological ileal loop model of intoxication. Future studies with animal models will determine whether PBA can be used to treat, as well as prevent, cholera.

## Materials and Methods

### Ethics statement

Ileal loop experiments were performed with approval from the Institutional Animal Care and Use Committee from the Center of Research and Advanced Studies (Centro de Investigacion y de Estudios Avanzados del IPN), protocol number 0364-07.

### Materials

The rabbit anti-CTA antibody, rabbit anti-CTB antibody, BfA, and ganglioside GM1 were purchased from Sigma-Aldrich (St. Louis, MO). CT was purchased from List Biological Laboratories (Campbell, CA), the purified CTA1/CTA2 heterodimer was purchased from Calbiochem (La Jolla, CA), and the His-tagged CTA1 constructs were purified in the laboratory as previously described [16]. Cell culture reagents were purchased from Invitrogen (Carlsbad, CA). [^35^S]methionine was purchased from Perkin-Elmer (Boston, MA). Rabbit anti-Hsp90 and anti-PDI antibodies were purchased from Stressgen Bioreagents Corp. (Victoria, BC Canada), and the horseradish peroxidase-conjugated goat anti-rabbit IgG antibody was from Jackson Immunoresearch Laboratories Inc. (West Grove, PA).

### CD and fluorescence spectroscopy

Thermal unfolding experiments were performed using a J-810 spectrofluoropolarimeter equipped with a PFD-425S Peltier temperature controller (Jasco Corp., Tokyo, Japan). The concentration of His-tagged CTA1 was 70 µg in 220 µL of 20 mM sodium borate buffer (pH 7.0) containing 100 mM NaCl. The concentration of the commercially available CTA1/CTA2 heterodimer was 73 µg in 220 µL of 20 mM sodium borate buffer (pH 7.0) containing 100 mM NaCl and, to reduce the heterodimer, 10 mM β-ME. Thermal unfolding was carried out in the temperature range of 18–60°C, and samples were allowed to equilibrate for 4 min at each temperature before measurement. For fluorescence spectra, CTA1 tryptophan residues were excited at 290 nm and the fluorescence emission was measured between 300 and 400 nm. CD spectra were recorded from 190 to 320 nm, which covers both near-UV and far-UV range and thus allowed us to detect thermal changes in both tertiary and secondary structures. Each spectrum was averaged from 5 scans. The observed ellipticity was converted to mean residue molar ellipticity, [θ], in units of degrees×cm^2^×dmol^−1^ using:
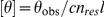
where θ_obs_ is the measured ellipticity in millidegrees, *c* is the molar concentration of the protein, *n_res_* is the number of amino acid residues in the protein, and *l* is the optical path-length in millimeters. Fluorescence and CD measurements were taken sequentially on the same sample in a 0.4 cm optical path-length quartz cuvette in order to reduce variability in sample-to-sample recordings. The temperature-dependent unfolding data were analyzed as previously described [11].

### SPR

Experiments were performed with a Reichert (Depew, NY) SR7000 SPR refractometer. The Reichert Labview software was used for data collection, and the BioLogic (Campbell, Australia) Scrubber 2 software was used for data analysis. For experiments using His-tagged CTA1, sensor slides were prepared as previously described [11]. For experiments using the CT holotoxin or the CTB pentamer, a gold plate sensor was coated with the GM1 ganglioside receptor of CT by a procedure described for the coating of ELISA plates [47]. CT or CTB_5_ was then bound to the GM1-coated sensor by perfusing 1 ml of toxin (10 µg/ml) over the slide for 15 minutes at a flow rate of 5 µl/min.

For experiments involving antibody-coated plates, an EDC-NHS activation buffer was perfused over a gold-plated glass slide for 10 min. A 5 min wash with 10 mM phosphate buffered saline containing 0.05% Tween 20 (PBST) was used to remove the activation buffer, after which an anti-CTA antibody at 1∶2000 dilution in PBST was perfused over the slide for 15 min. Unbound antibody was removed with a 5 min PBST wash, and the remaining active groups on the sensor slide were deactivated with a 3 min exposure to ethanolamine. Following each experimental condition, bound ligand was removed from the sensor slide with 0.01% imidazole in PBST at pH 5.5.

The following equation was used to determine the stoichiometry of CT/CTA1-PBA interactions:
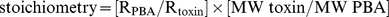
where R_PBA_ is the observed response of bound PBA, R_toxin_ is the response of surface-immobilized toxin, and MW is molecular weight [48].

### Translocation assays

For the translocation assay with exogenously applied CT, HeLa cells were seeded to 6-well plates in complete DMEM medium to achieve an 80% confluent monolayer after an overnight incubation at 37°C. Triplicate wells were required for each condition. To begin the experiment, cells were incubated with DMEM containing 100 ng/ml of GM1 for 1 h at 37°C. After washing, the cells were subsequently incubated for 30 min at 4°C with DMEM containing 1 µg/ml of CT. After further washing, the cells were returned to 37°C for 2 h in DMEM containing no additions, 100 µM PBA, or 5 µg/ml of BfA. The cells were then lifted from the 6-well plate using 750 µl of 0.5 mM ethylenediaminetetraacetic acid in phosphate buffered saline. All cells for each condition were collected in a single microcentrifuge tube and spun at 5,000× g for 5 min. The supernatant was discarded and the pellet was resuspended in 1 ml (SPR assay) or 0.1 ml (Western blot analysis) HCN buffer (50 mM Hepes pH 7.5, 150 mM NaCl, 2 mM CaCl_2_, 10 mM N-ethylmaleimide, and a protease inhibitor cocktail) containing 0.04% digitonin. After 10 min on ice, the samples were spun at 16,000× g for 10 min. Both supernatant (i.e,. cytosol) and pellet (i.e., membrane) fractions were collected and placed in fresh microcentrifuge tubes. For SPR analysis, supernatant samples were brought to a final volume of 1 ml in HCN buffer and perfused over a sensor slide coated with an anti-CTA antibody. For Western blot analysis, 120 µl of 1× sample buffer was added to the pellet and 20 µl of 4× sample buffer was added to the supernatant. Samples were resolved by SDS-PAGE with 15% polyacrylamide gels, and separate blots were run for each protein. The anti-Hsp90 antibody was used at a 1∶20,000 dilution, the anti-PDI antibody was used at a 1∶5,000 dilution, and the horseradish peroxidase-conjugated goat anti-rabbit IgG antibody was used at a 1∶20,000 dilution.

For the translocation assay with plasmid-expressed CTA1, CHO cells seeded to 80% confluency in 6-well plates were transfected with pcDNA3.1/ssCTA1 [49] using Lipofectamine (Invitrogen) according to the manufacturer's instructions. At 24 h post-transfection, cells were incubated in methionine-free medium for 1 h. [^35^S]methionine was then added for another hour. Where indicated, 100 µM PBA was present during both the methionine starvation and the radiolabeling. Both membrane and cytosolic fractions from digitonin-permeabilized cells were immunoprecipitated with an anti-CTA antibody. SDS-PAGE with PhosphorImager analysis was used to visualize and quantify the immunoisolated material. The extent of CTA1 translocation was calculated with the following equation:



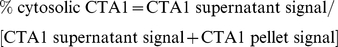
The percentage obtained from untreated control cells was set at 100%, and the percentage obtained from PBA-treated cells was expressed as a fraction of this value. Previous work has demonstrated this assay can detect the inhibition of CTA1 translocation in cells lacking Hsp90 function [38].

### Trafficking assay

HeLa cells were exposed to GM1 and CT as described above for the translocation assay. Where indicated, 100 µM PBA or 5 µg/ml of BfA was present in the 37°C chase medium. Membrane fractions from digitonin-permeabilized cells were resolved by non-reducing SDS-PAGE with 15% polyacrylamide gels. Western blot analysis was performed with a rabbit anti-CTA antibody (1∶20,000 dilution) and a horseradish peroxidase-conjugated goat anti-rabbit IgG secondary antibody (1∶20,000 dilution). In the commercially available CT holotoxin, a substantial fraction of CTA1 is already in a reduced state (unpublished observations). Thus, for this experiment, we used a recombinant CT holotoxin produced in *Escherichia coli*. The reduced form of CTA1 is initially absent in this recombinant toxin, so it was possible to clearly detect the reduction of the CTA1/CTA2 disulfide bond which occurs in the ER.

### Toxicity assays

HeLa cells were seeded to 24 well plates and grown overnight to 80% confluency. After a 1 h 37°C incubation with DMEM containing 100 ng/ml of GM1, the cells were challenged with various concentrations of CT in the absence or presence of PBA for 2 h at 37°C. The cells were then washed and exposed to 0.25 ml of ice-cold HCl∶EtOH (1∶100) for 15 min on ice. Cell extracts were placed in microcentrifuge tubes and allowed to air dry overnight. cAMP levels were then determined using an ELISA cAMP competition assay kit as per manufacturer (GE Healthcare, Piscataway, NJ) instructions. Background cAMP values obtained from unintoxicated cells were subtracted from all experimental values. The maximum cAMP response for the experiment was set to 100%, and all other values were expressed as a ratio of that value.

Ileal loop experiments were performed as previously described [38] after approval from the CINVESTAV-IPN Animal Ethical Committee. For studies involving peritoneal injection of PBA, the rats were injected 40 minutes before laparatomy with 100 µl of PBS or 100 µl of 0.18 M PBA. Injected rats were anesthetized with Xylacin (6.5 mg/Kg) and Ketamin (34.5 mg/Kg) to perform laparotomy and expose the small intestines. Four ileal loops of 3 cm with intervening gaps of 2–4 cm between them were then ligated 20 cm upstream of the ileocecal valve. The loops were injected with 200 µl of CT (3 µg/ml in PBS). Control loops from rats intraperitoneally inoculated with PBS were injected with 200 µl of PBS alone. After injection with CT or PBS, the loops were returned to the abdominal cavity and the incision was sutured. The rats were kept alive for 7 h before sacrifice by cervical dislocation. Ileal loops were photographed and dissected, and the intestinal contents were collected by gentle pressure. The volume of fluid in each loop was measured and expressed as a ratio of the amount (µl) of fluid per unit length (cm) of loop.

## Supporting Information

Figure S1 **PBA does not prevent the reductive separation of CTA1 from CTA2.** 1 µg samples of the CTA1/CTA2 heterodimer were exposed to 10 mM β-ME for 5 min in the absence or presence of 100 µM PBA before loading on a non-reducing SDS-PAGE gel. 1 µg of a CTA1/CTA2 heterodimer that was not exposed to β-ME was also run on the gel. Samples were visualized by Coomassie staining, which does not detect the dissociated 5 kDa CTA2 subunit.
(TIF)

Figure S2 **PBA inhibits the thermal unfolding of CTA1-His_6_.** (A–F): The temperature-induced unfolding of CTA1-His_6_ in the absence (A–C) or presence (D–F) of 100 µM PBA was monitored by near-UV CD (A, D), fluorescence spectroscopy (B, E), and far-UV CD (C, F). The change in color from blue to red corresponds to a change in temperature from 18°C to 60°C. (G–I): Thermal unfolding profiles for CTA1-His_6_ in the absence (red) or presence (blue) of 100 µM PBA were derived from the data presented in panels A–F. (G): For near-UV CD analysis, the mean residue molar ellipticities at 280 nm ([θ]_280_) were plotted as a function of temperature. *T*_m_ values of 33°C and 36°C were recorded for CTA1-His_6_ in the absence and presence of PBA, respectively. (H): For fluorescence spectroscopy, the maximum emission wavelength (λ_max_) was plotted as a function of temperature. *T*_m_ values of 35.5°C and 39°C were recorded for CTA1-His_6_ in the absence and presence of PBA, respectively. (I): For far-UV CD analysis, the mean residue molar ellipticities at 220 nm ([θ]_220_) were plotted as a function of temperature. *T*_m_ values of 35°C and 45°C were recorded for CTA1-His_6_ in the absence and presence of PBA, respectively.
(TIF)

Figure S3 **PBA does not inhibit the thermal unfolding of a CTA1 construct lacking the A1_3_ subdomain, CTA1_1–168_•His_6_.** (A–F): The temperature-induced unfolding of CTA1_1–168_·His_6_ in the absence (A–C) or presence (D–F) of 100 µM PBA was monitored by near-UV CD (A, D), fluorescence spectroscopy (B, E), and far-UV CD (C, F). The change in color from blue to red corresponds to a change in temperature from 18°C to 60°C. (G–I): Thermal unfolding profiles for CTA1_1–168_·His_6_ in the absence (red) or presence (blue) of 100 µM PBA were derived from the data presented in panels A–F. (G): For near-UV CD analysis, the mean residue molar ellipticities at 280 nm ([θ]_280_) were plotted as a function of temperature. *T*_m_ values of 32.5°C and 33°C were recorded for CTA1_1–168_·His_6_ in the absence and presence of PBA, respectively. (H): For fluorescence spectroscopy, the maximum emission wavelength (λ_max_) was plotted as a function of temperature. *T*_m_ values of 34°C and 33.5°C were recorded for CTA1_1–168_·His_6_ in the absence and presence of PBA, respectively. (I): For far-UV CD analysis, the mean residue molar ellipticities at 220 nm ([θ]_220_) were plotted as a function of temperature. *T*_m_ values of 36°C and 35.5°C were recorded for CTA1_1–168_·His_6_ in the absence and presence of PBA, respectively.
(TIF)

Figure S4 **Partial unfolding of reduced CTA1/CTA2 is a reversible process.** Fluorescence measurements were conducted on a reduced CTA1/CTA2 heterodimer. CTA1 tryptophan residues were excited at 290 nm and the fluorescence emission was measured between 300 and 400 nm. The simulated curve for a reduced CTA1/CTA2 heterodimer heated from 18°C to 65°C was used to fit the experimental data in both A and B. (A) Temperature dependence of the maximum emission wavelength of tryptophan fluorescence when reduced CTA1/CTA2 was heated from 18°C to 65°C (filled circles) and then cooled from 65°C to 18°C (open circles). (B) Temperature dependence of the maximum emission wavelength of tryptophan fluorescence when reduced CTA1/CTA2 was heated from 18°C to 37°C (filled circles) and then cooled from 37°C to 18°C (open circles).
(TIF)

Figure S5 **Calculation of cytosolic CTA1 from SPR-based translocation assays.** The association rate constants for the CTA standards from Fig. 4B were plotted as a function of protein concentration (closed circles). The association rate constants for CTA1 obtained from the cytosol of untreated (open circle) or PBA-treated (open square) cells were then plotted on the standard curve. A CTA1 concentration of 0.24 ng/ml was calculated for untreated cells, and a CTA1 concentration of 0.08 ng/ml was calculated for PBA-treated cells.
(TIF)

Figure S6 **Secretion of CTA1 from PBA-treated cells.** HeLa cells pulse-labeled at 4°C for 30 min with 1 µg/ml of CT were chased for 2 hr at 37°C in toxin-free medium that lacked (no treatment) or contained 100 µM PBA (+PBA). Media samples from these cells, from cells incubated with 5 µg/ml of BfA (+BfA), and from unintoxicated control cells were then analyzed by SPR with a sensor slide that had been coated with an anti-CTA antibody. CTA standards (10 ng/ml and 1 ng/ml) were also perfused over the sensor slide as positive controls. One of three representative experiments is shown. At the end of each experiment, bound sample was stripped from the sensor slide.
(TIF)
