# Supplementary figures and images for: A Therapeutic Chemical Chaperone Inhibits Cholera Intoxication and Unfolding/Translocation of the Cholera Toxin A1 Subunit
Source: PLoS One. 2011 Apr 19;6(4):e18825. doi: 10.1371/journal.pone.0018825 (PMC3079739; doi:10.1371/journal.pone.0018825)

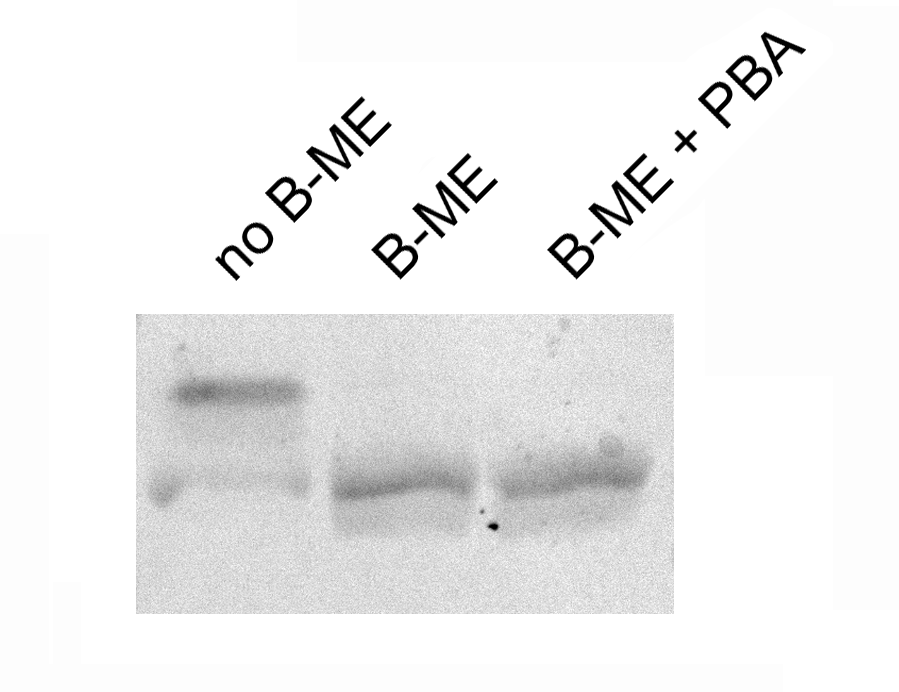

Supplement: Figure S1 — PBA does not prevent the reductive separation of CTA1 from CTA2. 1 µg samples of the CTA1/CTA2 heterodimer were exposed to 10 mM β-ME for 5 min in the absence or presence of 100 µM PBA before loading on a non-reducing SDS-PAGE gel. 1 µg of a CTA1/CTA2 heterodimer that was not exposed to β-ME was also run on the gel. Samples were visualized by Coomassie staining, which does not detect the dissociated 5 kDa CTA2 subunit. (TIF) [file pone.0018825.s001.tif]

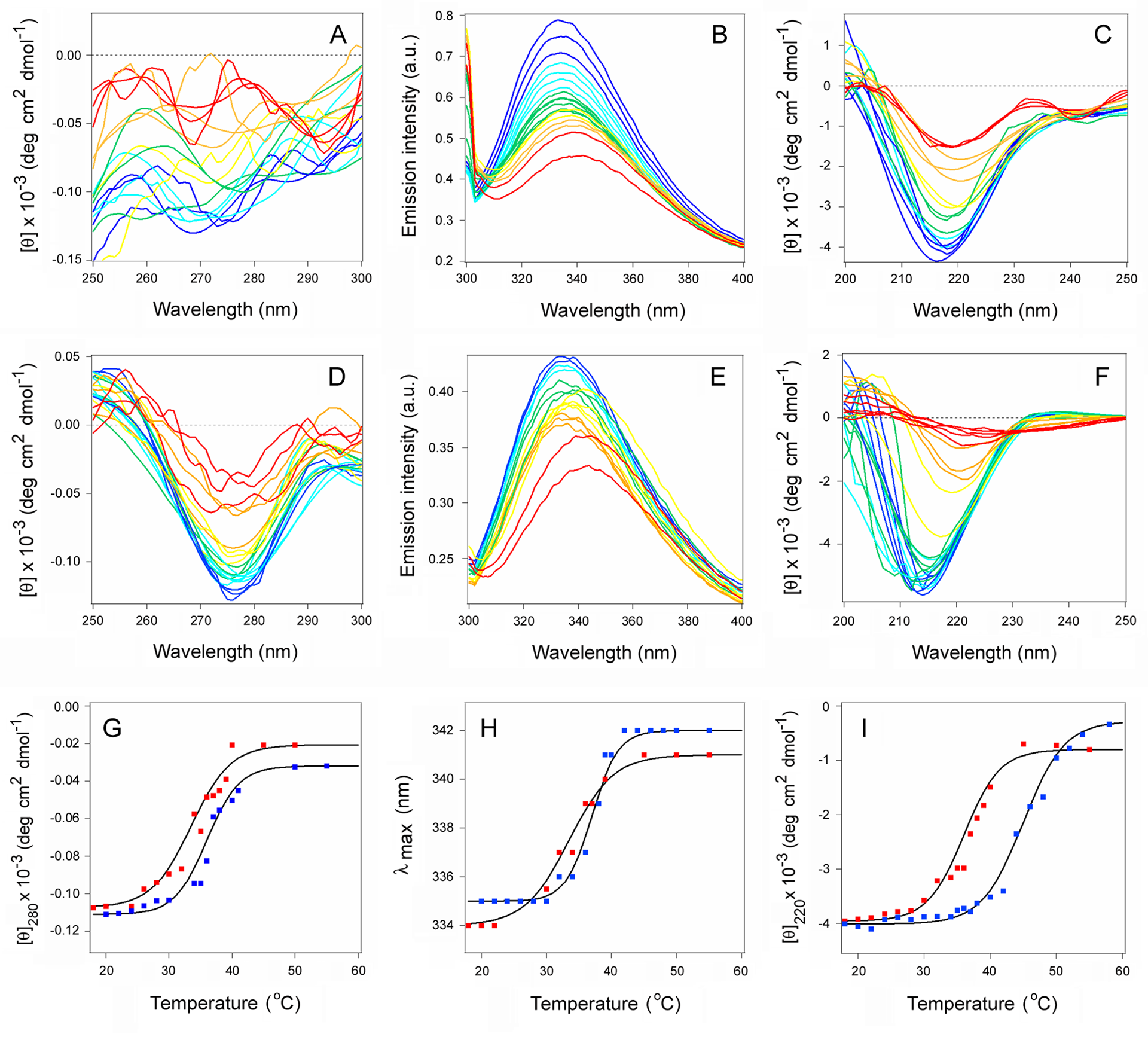

Supplement: Figure S2 — PBA inhibits the thermal unfolding of CTA1-His6. (A–F): The temperature-induced unfolding of CTA1-His6 in the absence (A–C) or presence (D–F) of 100 µM PBA was monitored by near-UV CD (A, D), fluorescence spectroscopy (B, E), and far-UV CD (C, F). The change in color from blue to red corresponds to a change in temperature from 18°C to 60°C. (G–I): Thermal unfolding profiles for CTA1-His6 in the absence (red) or presence (blue) of 100 µM PBA were derived from the data presented in panels A–F. (G): For near-UV CD analysis, the mean residue molar ellipticities at 280 nm ([θ]280) were plotted as a function of temperature. T m values of 33°C and 36°C were recorded for CTA1-His6 in the absence and presence of PBA, respectively. (H): For fluorescence spectroscopy, the maximum emission wavelength (λmax) was plotted as a function of temperature. T m values of 35.5°C and 39°C were recorded for CTA1-His6 in the absence and presence of PBA, respectively. (I): For far-UV CD analysis, the mean residue molar ellipticities at 220 nm ([θ]220) were plotted as a function of temperature. T m values of 35°C and 45°C were recorded for CTA1-His6 in the absence and presence of PBA, respectively. (TIF) [file pone.0018825.s002.tif]

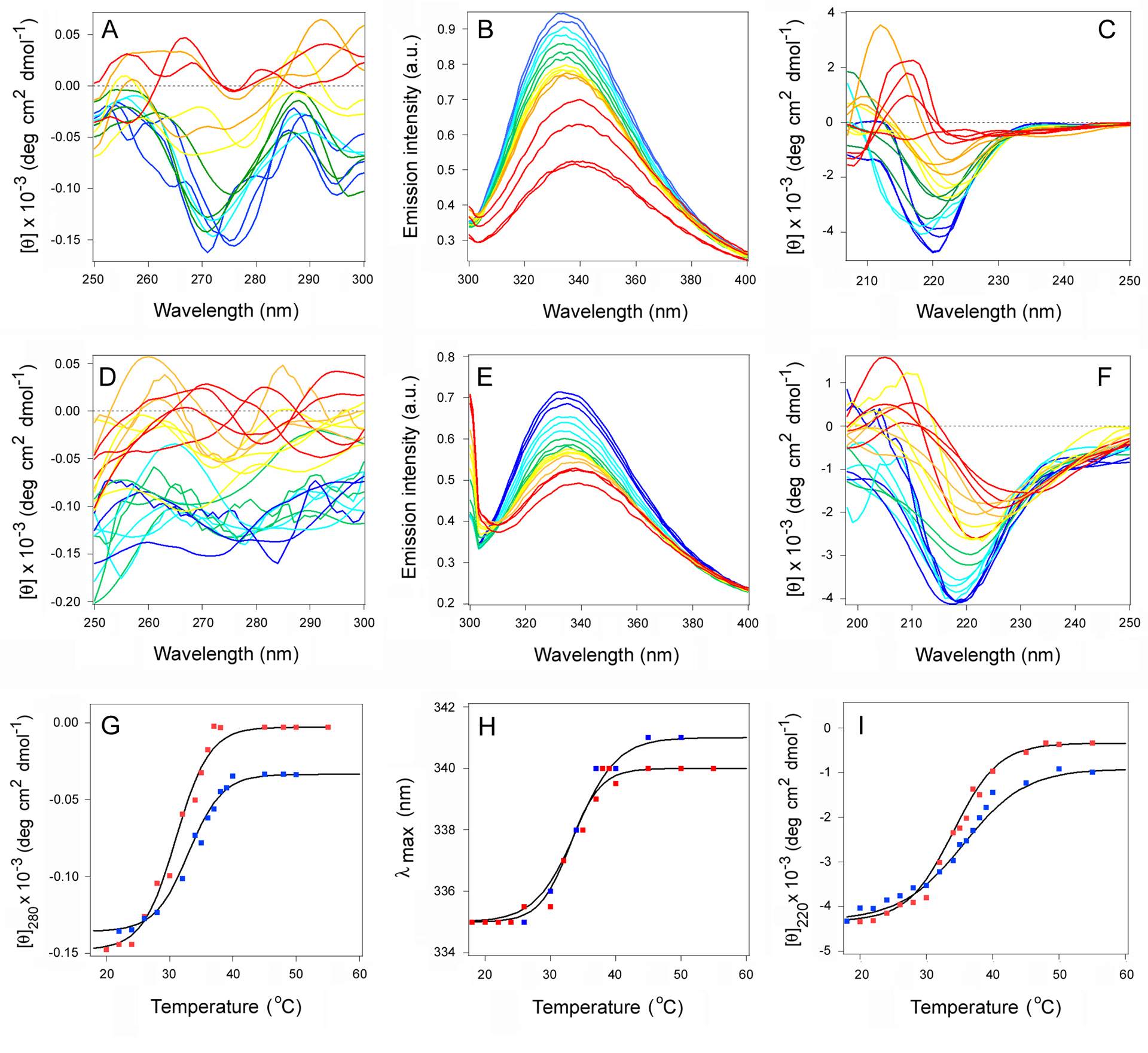

Supplement: Figure S3 — PBA does not inhibit the thermal unfolding of a CTA1 construct lacking the A13 subdomain, CTA11–168•His6. (A–F): The temperature-induced unfolding of CTA11–168·His6 in the absence (A–C) or presence (D–F) of 100 µM PBA was monitored by near-UV CD (A, D), fluorescence spectroscopy (B, E), and far-UV CD (C, F). The change in color from blue to red corresponds to a change in temperature from 18°C to 60°C. (G–I): Thermal unfolding profiles for CTA11–168·His6 in the absence (red) or presence (blue) of 100 µM PBA were derived from the data presented in panels A–F. (G): For near-UV CD analysis, the mean residue molar ellipticities at 280 nm ([θ]280) were plotted as a function of temperature. T m values of 32.5°C and 33°C were recorded for CTA11–168·His6 in the absence and presence of PBA, respectively. (H): For fluorescence spectroscopy, the maximum emission wavelength (λmax) was plotted as a function of temperature. T m values of 34°C and 33.5°C were recorded for CTA11–168·His6 in the absence and presence of PBA, respectively. (I): For far-UV CD analysis, the mean residue molar ellipticities at 220 nm ([θ]220) were plotted as a function of temperature. T m values of 36°C and 35.5°C were recorded for CTA11–168·His6 in the absence and presence of PBA, respectively. (TIF) [file pone.0018825.s003.tif]

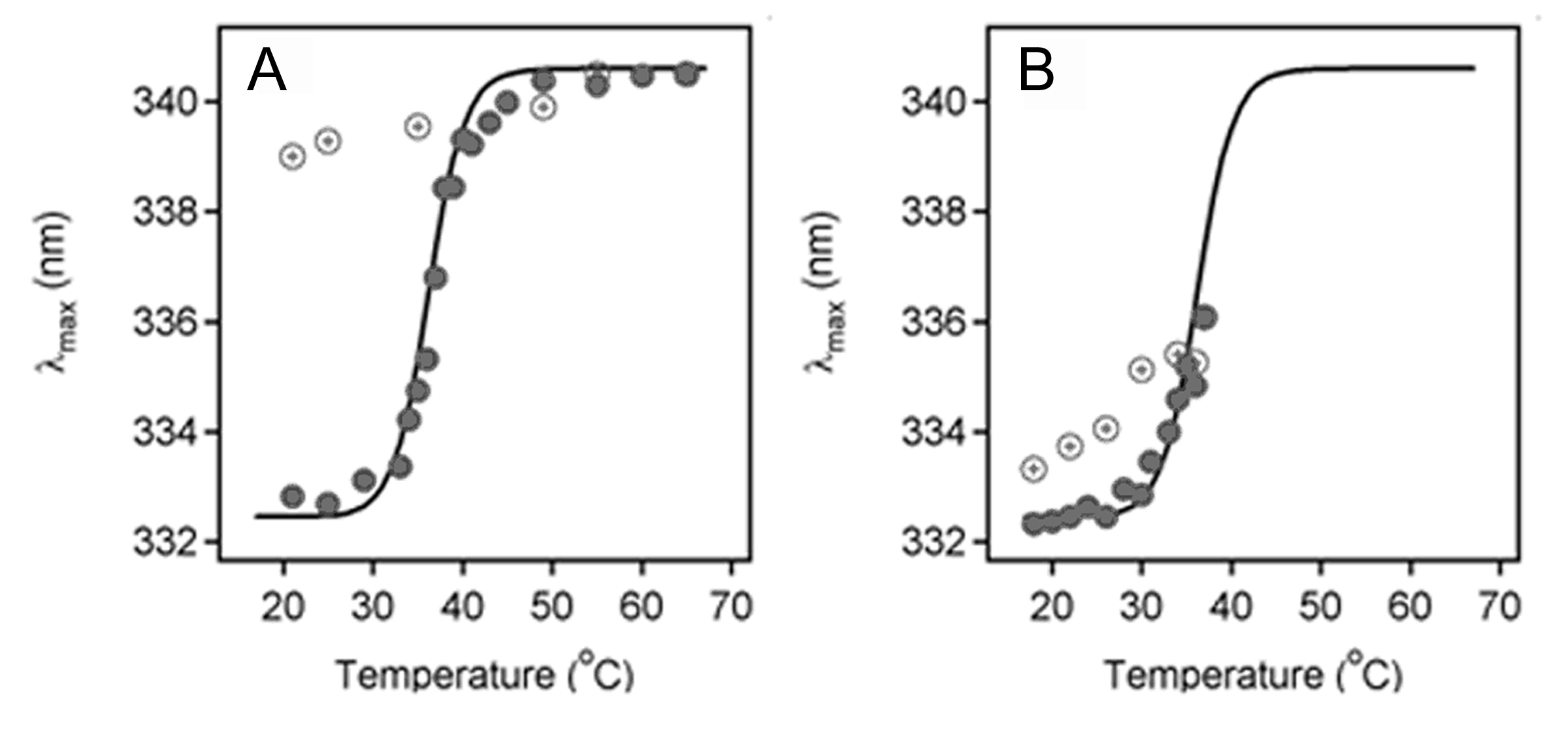

Supplement: Figure S4 — Partial unfolding of reduced CTA1/CTA2 is a reversible process. Fluorescence measurements were conducted on a reduced CTA1/CTA2 heterodimer. CTA1 tryptophan residues were excited at 290 nm and the fluorescence emission was measured between 300 and 400 nm. The simulated curve for a reduced CTA1/CTA2 heterodimer heated from 18°C to 65°C was used to fit the experimental data in both A and B. (A) Temperature dependence of the maximum emission wavelength of tryptophan fluorescence when reduced CTA1/CTA2 was heated from 18°C to 65°C (filled circles) and then cooled from 65°C to 18°C (open circles). (B) Temperature dependence of the maximum emission wavelength of tryptophan fluorescence when reduced CTA1/CTA2 was heated from 18°C to 37°C (filled circles) and then cooled from 37°C to 18°C (open circles). (TIF) [file pone.0018825.s004.tif]

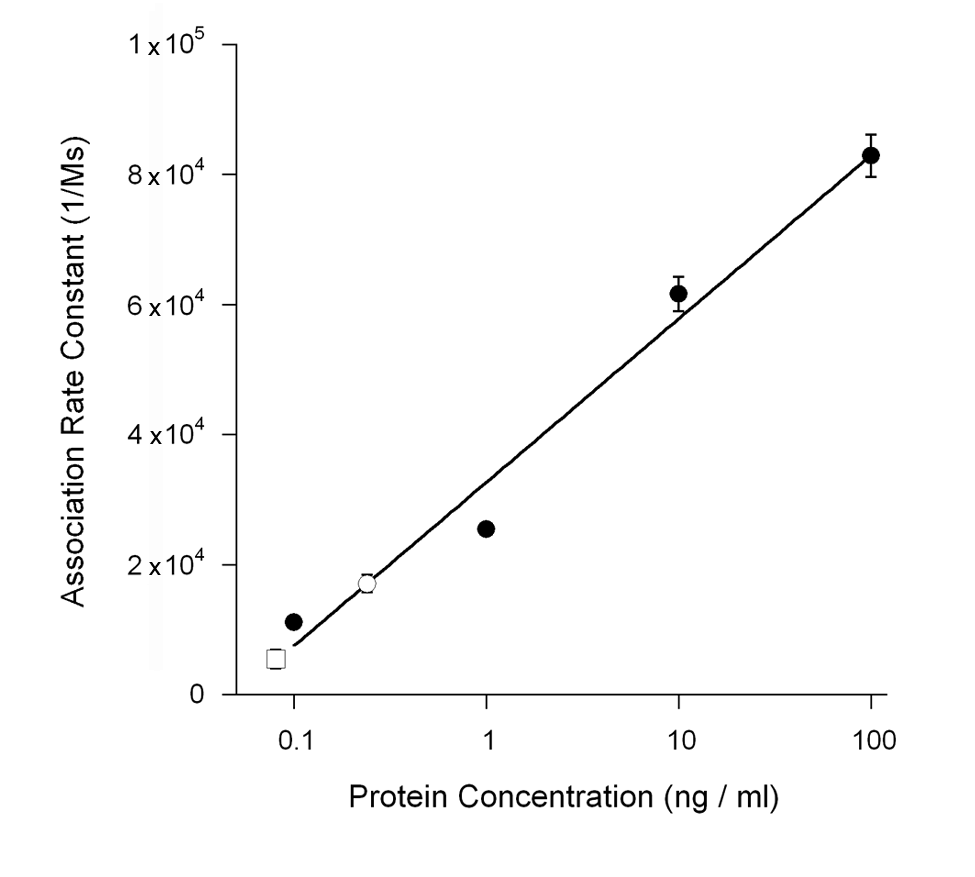

Supplement: Figure S5 — Calculation of cytosolic CTA1 from SPR-based translocation assays. The association rate constants for the CTA standards from Fig. 4B were plotted as a function of protein concentration (closed circles). The association rate constants for CTA1 obtained from the cytosol of untreated (open circle) or PBA-treated (open square) cells were then plotted on the standard curve. A CTA1 concentration of 0.24 ng/ml was calculated for untreated cells, and a CTA1 concentration of 0.08 ng/ml was calculated for PBA-treated cells. (TIF) [file pone.0018825.s005.tif]

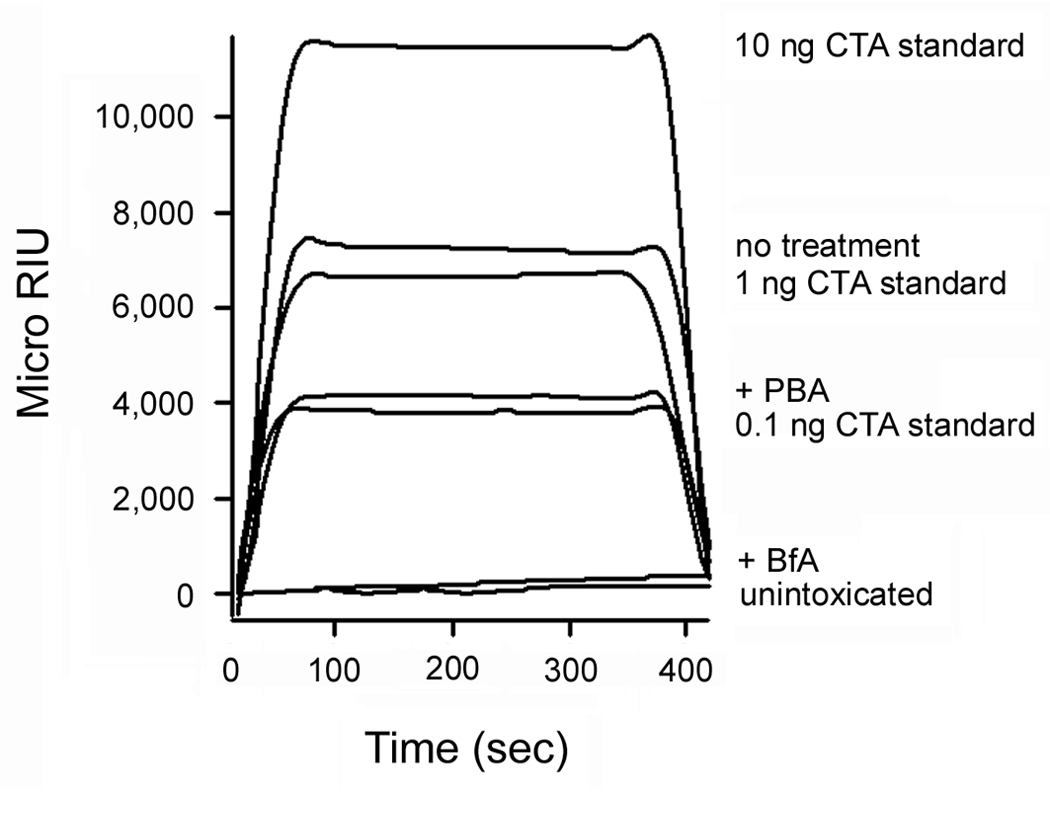

Supplement: Figure S6 — Secretion of CTA1 from PBA-treated cells. HeLa cells pulse-labeled at 4°C for 30 min with 1 µg/ml of CT were chased for 2 hr at 37°C in toxin-free medium that lacked (no treatment) or contained 100 µM PBA (+PBA). Media samples from these cells, from cells incubated with 5 µg/ml of BfA (+BfA), and from unintoxicated control cells were then analyzed by SPR with a sensor slide that had been coated with an anti-CTA antibody. CTA standards (10 ng/ml and 1 ng/ml) were also perfused over the sensor slide as positive controls. One of three representative experiments is shown. At the end of each experiment, bound sample was stripped from the sensor slide. (TIF) [file pone.0018825.s006.tif]
